# Nutrition Information Briefs – Niacin^☆^

**DOI:** 10.1016/j.advnut.2026.100646

**Published:** 2026-05-08

**Authors:** Mirella L Meyer-Ficca, Claudia CS Chini

**Affiliations:** 1 Department of Veterinary Clinical and Life Sciences, College of Veterinary Medicine, Utah State University, Logan, UT, United States; 2 Department of Anesthesiology and Perioperative Medicine, Metabolism and Molecular Nutrition Laboratory, Kogod Center on Aging, Mayo Clinic College of Medicine, Jacksonville, FL, United States

**Keywords:** aging, DNA repair, metabolism, mitochondria, poly(ADP-ribose)polymerase, pellagra, redox reaction, sirtuin, vitamin B3 (niacin, nicotinamide, nicotinamide ribose, nicotinamide mononucleotide), NAD (nicotinamide adenine dinucleotide)

## Niacin

Niacin is an essential precursor for the synthesis of NAD and for its phosphorylated derivative, NADP. Both pools undergo 2-electron redox reactions, generating distinct redox couples (NAD^+^/NADH and NADP^+^/NADPH) that serve as coenzymes for distinct enzymatic reactions in metabolism [1]. In healthy cells and tissues, the NAD pool is maintained predominantly in a highly oxidized state (∼90% NAD^+^), mainly through the activity of the electron transport chain.

Conversely, the NADP pool is maintained mostly in the reduced state, mainly through the pentose phosphate pathway. The result is that enzymes linked to the NAD redox couple become strongly oxidizing, whereas those linked to the NADP redox couple become strongly reducing. Remarkably, the NAD redox couple participates in ∼400 reactions, predominantly in catabolic pathways involved in energy production, such as glycolysis, the citric acid cycle, fatty acid oxidation, and ethanol oxidation. In contrast, the NADP redox couple participates in ∼30 reactions, largely supporting anabolic metabolism including fatty acid and cholesterol synthesis, as well as oxidant defense and detoxification pathways, such as cytochrome P450-mediated metabolism of endo- and xenobiotics. In addition to redox-dependent reactions, more than 2 dozen ADP-ribosyl transferases and hydrolases use NAD^+^ as a substrate, leading to its degradation. Some of these enzymes add poly(ADP-ribose) chains to DNA repair enzymes or mono ADP-ribose units to various proteins, thereby regulating their activity. For example, NAD-dependent deacetylases such as sirtuin proteins (involved in metabolic energy sensing and epigenetic control) use ADP-ribose as an acceptor to deacetylate substrates. Lastly, NAD-derived products cyclic ADP-ribose and nicotinic acid-ADP (NAADP) act to control intracellular calcium signaling, critical to nervous system function. Many of these roles are reflected by the pathologies of niacin deficiency.

## Deficiencies

Severe niacin deficiency presents in humans as the disease pellagra, which is characterized by the “4 Ds” (dermatitis, dementia, diarrhea, and ultimately death). The photosensitive nature of the dermatitis—known as “Casal’s necklace”—suggests a defect in DNA repair, and is now thought to be due to impaired synthesis of poly ADP-ribose in response to UV-radiation induced DNA damage. Numerous experimental models have demonstrated genomic instability in response to niacin deficiency. Recent human epidemiologic studies further point toward a negative correlation between niacin intake and tumor incidence [2]. Pellagra-associated dementia is also unique: while it presents similar to schizophrenia with hallucinations and delusions, it responds within hours to niacin therapy. The symptoms are thought to be due to impaired formation of cyclic ADP-ribose and NAADP, leading to altered neural calcium signaling.

Malnutrition-associated pellagra has been largely eliminated in developed countries by niacin fortification of flour, but subclinical deficiencies of niacin persist, and their relationship to disease susceptibility is understudied. Congenital NAD deficiency disorders, such as Hartnup disease and mutations affecting NAD synthesis and consumption pathways, are rare. More common causes of niacin deficiencies include malabsorption, alcoholism, drug treatments, anorexia nervosa and malignancies. In experimental models, niacin supplementation has demonstrated benefits across a range of conditions, including cancer, cardiovascular disease, skin health, mental health, and oxidant-induced lung injury.

## Diet Recommendations

Recommended dietary allowances (RDA) and tolerable upper intake levels (UL) for niacin equivalents (NE) [3] are summarized in Table 1 [3].

**TABLE 1:** RDA and UL for NE [3]

Age (y)	0–0.5	0.5–1	1–3	4–8	9–13	≥14	Pregnancy	Lactation
RDA (mg of niacin for age 0–0.5 y, NE for other age groups)	2 (AI)	4 (AI)	6	8	12	♀ 4 ♂ 16	18	17
UL (mg of supplemental and fortified niacin)	ND	ND	10	15	20	30–35	30–35	30–35

Abbreviations: ND, not determined; NE, niacin equivalents; RDA, recommended dietary allowances; UL, tolerable upper intake levels.

## Food Sources

Besides nicotinamide and nicotinic acid, the 2 forms of niacin, tryptophan can be converted to NAD with low efficiency, leading to the concept of NE (= mg niacin + 1/60th mg tryptophan). Fish and meats (beef, poultry) are excellent sources of NE, due to high levels of tryptophan and NAD/NADP that releases nicotinamide during digestion. Nuts and legumes are also good sources of NE, due to a combination of nicotinic acid and tryptophan, combined with their high level of intake. Cow’s milk is rich in nicotinamide riboside (NR), another NAD precursor [4]. Lastly, niacin fortification in food is common in most developed countries, adding approximately 1 to 16 mg per serving in flour and cereal products, and up to 40 mg in energy drinks. Conversely, corn is a poor source of NE because it contains little tryptophan and its niacin is tightly bound, requiring alkaline food processing to make it bioavailable. Such food processing traditions were lost when corn was discovered in the Americas and distributed to the rest of the world, resulting in pellagra epidemics in corn-eating populations.

## Clinical Uses

Niacin supplementation is the classical and only Food and Drug Administration–approved treatment for pellagra, which is rare in developed countries. Although early clinical trials showed that pharmacological doses of nicotinic acid (1–3 g/d) reduce serum LDL cholesterol and increase serum HDL cholesterol, these effects did not translate into consistent clinical benefits for the prevention and general treatment of cardiovascular diseases (CVDs). This resulted in a shift toward more specific indications, such as lowering lipoprotein (a) in patients with a genetic risk for heart disease or treating severe hypertriglyceridemia in patients that are not responsive to statin, and to manage elevated lipoprotein (a) concentrations and hyperphosphatemia in end-stage renal disease patients on hemodialysis. The use of nicotinamide as a skin cancer chemopreventative in dermatological clinics appears to be supported by a large scale retrospective study, although there is not yet consensus in the medical community regarding its efficacy [5,6].

## Toxicity

Toxicity from niacin is rare and does not occur with niacin derived from natural food sources or from tryptophan. The UL for nicotinic acid (35 mg/d in adults in the United States, 10 mg/d in Europe) is based primarily on the skin flushing reaction frequently seen with excessive intake of nicotinic acid from supplements and food fortification. At higher doses, niacin is associated with liver toxicity.

Mechanistically, nicotinic acid appears to act as a nonphysiological ligand for various cellular receptors on adipocytes and skin cells. Activation of these receptors is thought to trigger arachidonic acid release through prostaglandin signaling pathways, contributing to the skin-flushing and vasodilation. This response generates uncomfortable heat and itching but is not considered to pose a long-term risk or lead to pathologies, and often diminishes with repeated exposure. Nicotinic acid receptors are also involved in metabolic sensing, modulating fatty acid release, and prostaglandin synthesis. In humans, the flushing response can be only partially controlled with prostaglandin synthesis inhibitors, suggesting the involvement of additional niacin receptors.

Of interest, pure niacin supplements are generally formulated at doses ranging from 100 to 500 mg, and B50, B75, and B100 vitamin combinations all exceed the UL for nicotinic acid, which is a regulatory anomaly. Recommended safe levels for other NAD precursors are higher, with up to 900 mg nicotinamide or 300 mg/d NR chloride considered safe for healthy adults [[7], [8], [9]]. In addition to the well-known risk of liver toxicity, recent studies indicate that high dose niacin supplementation increases the incidence of type 2 diabetes and that niacin degradation products are associated with increased inflammation-associated adverse cardiovascular events [10,11]. These findings are particularly relevant given the current trend of promoting niacin as an antiaging supplement.

## Recent Research

NAD tissue levels decline with age [12], and its central role in energy metabolism and mitochondrial function is emerging as a common underlying theme in clinical trials targeting various age-associated conditions and chronic diseases. Ongoing clinical trials are focusing on the anti-inflammatory, metabolic, and mitochondrial properties of NAD, testing NAD-boosting strategies using niacin, NR, or nicotinamide mononucleotide. These interventions are being evaluated for benefits in muscle function and physical endurance, sleep, mitochondrial myopathies, metabolic diseases such as prediabetes and diabetes, diabetes-related kidney diseases, and neurodegenerative disorders like Alzheimer’s and Parkinson’s Disease, and glaucoma [13]. Additional trials are examining the benefits of vitamin B3 on female infertility, and outcomes in in vitro fertilization.

At the cellular level, important recent NAD-related studies have shown that cellular NAD is present in discrete subcellular pools that are controlled by different enzymes and have identified SLC25A51 as the transporter required for NAD transport into the mitochondria [14]. At the organismal level, studies demonstrated that the enzyme α-amino-β-carboxymuconate-ε-semialdehyde decarboxylase (ACMSD) in the de novo NAD synthesis pathway is a key regulator of systemic NAD levels [15,16].

Current research is frequently directed at the role of niacin and NAD in aging-related processes. The rationale for these studies is the intimate connection between dietary niacin intake and resulting NAD levels, and the observation that NAD levels decline with age. This has led to the concept that lower NAD levels contribute to human aging-associated pathologies by impairing NAD-dependent nuclear and mitochondrial functions [17]. Molecular mediators of such aging-related pathologies are NAD-dependent enzymes, e.g., poly(ADP-ribose) polymerase (PARP) enzymes, CD38, and sirtuin proteins. Noteworthy in this context are recent mechanistic studies linking the age-related NAD depletion to increased activity of the NAD-consuming enzyme CD38, and studies connecting NAD metabolism to the senescence-associated secretory phenotype (SASP) involved in the proinflammatory properties of aging cells [[18], [19], [20], [21], [22], [23]]. In contrast, NAD supplementation in rodent models was shown to reduce cellular senescence, ameliorate inappropriate inflammation and improve metabolic and mitochondrial health [24]. Another mechanistic link between low NAD levels and aging is the “epigenetic noise” concept. According to this concept, increased DNA damage during aging triggers increased activity of DNA-damage responsive PARP enzymes, which causes a decline in NAD levels. The lowered NAD levels in turn cause reduced sirtuin activity, resulting in a decrease in sirtuin-mediated epigenetic control of cellular gene expression, ultimately resulting in “epigenetic noise” with unintended gene expression changes and cellular senescence.

Supplementation with dietary NAD^+^ precursors counteracted various age-associated diseases in animal models, triggering trials to test the potential benefits of increasing NAD levels to prevent the age-associated health decline. In humans, health-span benefits of such NAD boosting measures could not yet be consistently and conclusively demonstrated. A promising recent study using mouse models and human data sets showed that pharmacologically stabilizing NAD homeostasis slowed and potentially reversed Alzheimer’s disease [25]. This aligns with a recently described bidirectional relationship between cellular NAD pools and mitophagy and with observations that NAD boosting activates mitophagy-mediated clearing of amyloid plaques in animal models [26,27]. A comparative clinical trial evaluating 3 NAD precursors, nicotinamide (NAM), nicotinamide riboside (NR), and nicotinamide mononucleotide (NMN), found that oral administration of NAM causes a fast but mild NAD increase, whereas NR and NMN had low immediate effects but led to a more pronounced and sustained NAD elevation over time. This sustained response appears to result from the slow release of NAM and nicotinic acid during metabolism by the gut microbiota [28]. Further research is needed to identify which supplementation strategies using nutritional NAD precursors are most suitable for boosting NAD levels, and to ultimately evaluate the potential antiaging benefits associated with vitamin B3. For further information refer to references [1,3,12,17].

## Author contributions

M.L.M-F and C.C.C. both participated in writing, have read and approved the final manuscript.

## Funding

This work was supported by the Eunice Kennedy Shriver National Institute of Child Health and Human Development of the National Institutes of Health under award number HD103027 to M.L.M.-F.
